# Impact of Environmental Sub-Inhibitory Concentrations of Antibiotics, Heavy Metals, and Biocides on the Emergence of Tolerance and Effects on the Mutant Selection Window in *E. coli*

**DOI:** 10.3390/microorganisms11092265

**Published:** 2023-09-09

**Authors:** Kelechi B. Chukwu, Ovokeroye A. Abafe, Daniel G. Amoako, Arshad Ismail, Sabiha Y. Essack, Akebe L. K. Abia

**Affiliations:** 1 Antimicrobial Research Unit, College of Health Sciences, University of KwaZulu-Natal, Durban 4000, South Africa; kelechi@skblogistics.com (K.B.C.); o.abafe@bham.ac.uk (O.A.A.); amoakodg@gmail.com (D.G.A.); 2 Residue Laboratory, Agricultural Research Council, Onderstepoort Veterinary Research Campus, Onderstepoort 0110, South Africa; 3 School of Geography, Earth and Environmental Sciences, University of Birmingham, Birmingham B15 2TT, UK; 4 Department of Integrative Biology and Bioinformatics, University of Guelph, Guelph, ON N1G 2W1, Canada; 5 Sequencing Core Facility, National Institute for Communicable Diseases, National Health Laboratory Service, Johannesburg 2192, South Africa; arshadi@nicd.ac.za; 6 Department of Biochemistry and Microbiology, University of Venda, Thohoyandou 0950, South Africa; 7 Environmental Research Foundation, Westville 3630, South Africa

**Keywords:** environmental stressors, antibiotic resistance, selection pressure, public health, tolerant bacteria, environmental pollution, mutation, single nucleotide polymorphisms

## Abstract

Bacteria’s ability to withstand the detrimental effects of antimicrobials could occur as resistance or tolerance with the minimum inhibitory concentration, the mutant prevention concentration, and the mutant selection window as salient concepts. Thus, this study assessed the impact of exposure to extremely high doses of ampicillin on the level of persistence and tolerance development in isolates previously exposed to different concentrations of selected antibiotics, biocides, and heavy metals. These isolates were previously exposed to oxytetracycline (OXYTET), amoxicillin (AMX), copper (Cu), zinc (Zn), benzalkonium chloride (BAC) 10, dimethylammonium chloride (DADMAC) 12 and a combination of all the individual pollutants (ALL). The isolates were exposed to very high concentrations (25 × MIC) of ampicillin, and their tolerance was calculated as the time required to kill 99.9% of the bacterial population (MDK_99.9_). The MDK_99.9_ increased by 30 to 50% in test isolates (DADMAC, OXYTET, Zinc = 28 h; BAC, Copper = 30 h; amoxycillin, ALL = 26 h) compared to the untreated control. BAC-exposed isolates decreased from 2.5 × 10^8^ CFU/mL to 2.5 × 10^4^ CFU/mL on the second day, displaying the highest tolerance increase. The tolerance appeared to originate from two sources, i.e., stochastic persistence and genetic-induced persistence, involving multiple genes with diverse mechanisms. The mutant selection window of the isolates to ampicillin, amoxicillin, and oxytetracycline also slightly increased compared to the control, indicating the selective survival of persister cells during the 30-day exposure. These findings indicate that bacterial exposure to sub-inhibitory concentrations of environmental chemical stressors may not always result in the development of antimicrobial resistance but could initiate this process by selecting persisters that could evolve into resistant isolates.

## 1. Introduction

Bacteria and other microorganisms have continuously adapted to adverse stressors originating from natural and anthropogenic activities in the environment. These adaptations are exhibited phenotypically through persistence, biofilm formation, resistance, and tolerance, and genotypically through the acquisition of resistance and tolerance genes.

Resistance is the ability of bacteria to survive, grow, and replicate in the presence of antimicrobials at concentrations beyond the minimum inhibitory concentrations (MIC); this usually manifests as direct drug inactivation, decreased intake, increased efflux of the drug, and the alteration of the drugs’ binding sites [1,2,3]. Tolerance, on the other hand, is the ability of the bacteria to survive low to extremely high antimicrobial concentrations, usually above bactericidal concentrations but without growth [2]. Tolerance occurs through mechanisms such as dormancy, reduced metabolism, oxidative stress, and adenosine triphosphate (ATP) level maintenance. On their part, persisters are a naturally occurring sub-population of bacteria, making up about 0.000001% of the overall population. Their lack of growth makes them non-susceptible to antimicrobials and other environmental stressors, favouring their survival in the presence of extremely high pressure from these stressors [4]. Unlike resistance, which involves a one-gene-one-phenotype expression, persistence and tolerance are associated with multiple genes [5,6,7,8].

Furthermore, while resistance increases the MIC of the mutants compared with the susceptible parental strains, the MIC of the parental and the evolved strains remain the same during tolerance. Contrarily, tolerance increases the MDK_99,_ the minimum time required to kill 99% of the bacteria in a culture [9]. Generally, persister cells are genetically similar to parental and non-persister cells in a given bacterial population and have the same MIC. However, their presence is responsible for the biphasic killing pattern observed in bacteria, which usually starts exponentially with the killing of the susceptible cells, followed by the persister cells [5,9].

In addition to resistance and tolerance, another factor influencing microbial non-susceptibility to antimicrobials is the mutant selection window (MSW). The MSW represents the concentration range which allows the emergence of resistant mutants within a bacterial population. It is the range between the minimum inhibitory concentration (MIC) and the mutant prevention concentration (MPC) [10]. The MIC is the minimum concentration of an antimicrobial that inhibits bacterial growth, while the MPC is the threshold above which it is predicted that selection pressure would rarely lead to the proliferation of resistant mutants in the bacterial population [11]. The length of the MSW plays a crucial role in the selection of resistance in bacteria. The shorter the MSW, the smaller the drug concentration range required to eliminate the bacteria, and the better the chances of preventing the development of resistant mutants [12].

Effluents from hospital and manufacturing sites may contain chemical pollutants, including pharmaceuticals, especially antibiotics and heavy metals, at concentrations usually higher than the environmental values. For example, in Africa, sulfamethoxazole (SMX) has been detected at concentrations of 20.6 µg/L in hospital effluents compared to 6.8–7.8 µg/L in wastewater treatment plants and surface waters [13]. Also, ciprofloxacin (CIP) levels detected in industrial effluents were up to 31,000 µg/L, over 100 times the toxic level of most bacteria [13,14,15]. Karkman et al. [14] suggested that such high concentrations, above the bactericidal levels, were responsible for the emergence of antimicrobial resistance (AMR) in the environment. Although concentrations above the MPC rarely favour the emergence of resistant mutants [16,17], environmental concentrations of these stressors are not static, usually fluctuating between very low to extremely high levels, depending on the distance from the source and prevailing weather conditions. Such fluctuations could expose the bacteria to sub-inhibitory concentrations and contribute to the emergence of persister cells and the subsequent development of tolerance in the bacterial populations. Therefore, it is essential to investigate the effect of prolonged exposure to sub-bactericidal concentrations, as seen in the environment, on the emergence of tolerance to stressors in bacteria.

The standard technique to assess tolerance is through time-kill measurements, in which bacteria are exposed to an antimicrobial and the viable colony forming units (CFUs) are determined and plotted against time [18]. When the killing is exponential, the killing rate can be used to measure tolerance, which is the minimum duration of killing (MDK) at a certain percentile of the population; the percentile is expressed as an index in the MDK value. Therefore, MDK*_n_* is the minimum time required to kill *n*% of a bacterial population. Conversely, a high MDK suggests that killing the bacteria would require more time, corresponding to high tolerance [18]. Hence, tolerance is the ability of bacteria to stay alive even at bactericidal antimicrobial concentrations [19].

In our previous experiment, it was observed that the environmental concentrations of oxytetracycline (OXYTET), amoxicillin (AMX), copper (Cu), zinc (Zn), benzalkonium chloride (BAC) 10, dimethylammonium chloride (DADMAC) 12 and a combination of all the individual pollutants (ALL) could not elicit phenotypic or genotypic resistance in *E. coli* following exposure for 30 days [20]. Therefore, the current study assessed the impact of these exposures on the level of persistence and tolerance development in the exposed isolates, using an extremely high ampicillin concentration. The study further assessed the associations with observed mutations via whole genome sequencing (WGS) and single nucleotide polymorphisms (SNP) and investigated the impact of such exposure on the MSW of these exposed *E. coli* isolates to ampicillin, oxytetracycline, and amoxicillin. Although previous studies have demonstrated the development of resistance in bacteria following exposure to pharmaceuticals, such studies have mostly used unrealistically high concentrations, which would seldom be encountered in the environment [21,22]. Here, the concentrations used were those previously identified in the environment [23] to mimic real environmental conditions. It was hypothesised that exposure to environmental concentrations of biocides, antibiotics, and heavy metals induce tolerance in the exposed bacteria, increasing their MSW.

## 2. Materials and Methods

All chemical stressors used in this study were purchased from Sigma-Aldrich (Kempton Park, South Africa). The preparation of the standards for each chemical has previously been reported [23]. The concentrations used for the 30-day exposure experiments have also been published [20]. In the current study, two sets of experiments were conducted using the 30-day exposed isolates, first to determine the MSW to amoxicillin, oxytetracycline and ampicillin and then their MDK_99_._9_ when exposed to extremely high doses of ampicillin.

### 2.1. Test Organisms

The test organisms were the 30-day exposed *E. coli* (ATCC strain 25922) isolates from the earlier experiment [20], while wholly susceptible *E. coli* (ATCC strain 25922) were used as control. Bacteria inocula at concentrations of 1.5 × 10^8^ CFU/mL (McFarland standard) were prepared using the 30-day exposed isolates and the wholly susceptible *E. coli* (ATCC strain 25922).

### 2.2. Determination of Bacterial Tolerance

For this experiment, the time required to kill 99.99% of the isolates (MDK_99.99_) was estimated by modifying the method by Fridman et al. [24]. First, the exposed isolates were cultured overnight on nutrient agar at 37 °C to obtain fresh, viable, and concentrated isolates to monitor killing rates adequately. Then, the cultured isolates were scraped into 1 mL of Luria-Bertani (LB) broth (Merck Life Science (Pty) Ltd., Johannesburg, South Africa) and cultured overnight. After that, 1 mL of the concentrated culture was inoculated into 5 mL LB broth supplemented with 100 g/mL ampicillin (about 25 times the *E. coli* ampicillin MIC value). Each culture mixture was prepared in 2 × triplicate sets for three different time intervals (T_a_ = 3, 5, and 8 h), and incubated at 37 °C in a shaking incubator at 200 rpm.

After incubation and depending on the time interval (3, 5 and 8 h), the culture was washed twice in 5 mL of LBL by centrifuging at 1400× g for 10 min. Next, the pellets were resuspended in 1 mL of LBL and incubated for up to 24 h (21, 19 and 16 h, respectively) at 37 °C in a shaking incubator at 200 rpm. This gave a fresh 24 h culture for the following day’s exposures. After 24 h, the plate was cell counted and recorded. The overnight culture was then subjected to the same exposure as the previous day for three consecutive days, under the same experimental conditions. To confirm tolerance and not resistance, the surviving *E. coli* cells were also subjected to antimicrobial susceptibility testing (AST) using the broth microdilution method to ascertain changes in their MICs compared to the original stock [24].

### 2.3. Mutant Selection Window

The MSW is an antimicrobial concentration range between the MIC and MPC. The MIC was performed using the European Committee on Antimicrobial Susceptibility Testing (EUCAST) broth microdilution method [25,26,27]. Briefly, two-fold serial dilutions of the test agents (environmentally determined amoxicillin, ampicillin and oxytetracycline) were dispensed into microdilution plates and inoculated with *E. coli*. The plates were incubated at 37 °C for 24 h and read visually. The MPC was obtained by determining the MIC of a higher microbial load (≥10^9^ CFU/mL), as previously described [28].

To determine gene mutations that could lead to tolerance, isolates from all the experimental rounds were subjected to whole genome sequencing. All contiguous sequences for the isolates were deposited in GenBank with accession numbers under BioProject PRJNA836107.

## 3. Results

### 3.1. Determination of Tolerance

#### 3.1.1. Tolerance among Biocide-Exposed Isolates

**Figure 1:**
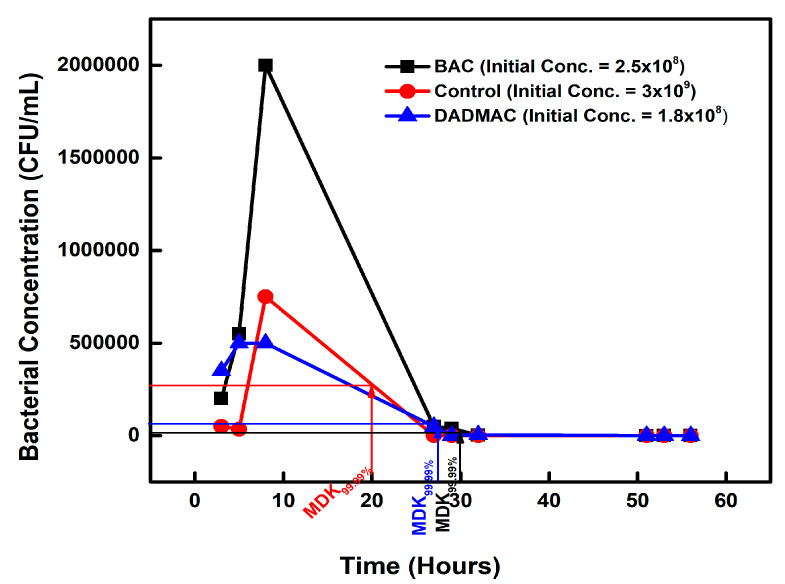
The minimum duration for killing 99.99% (MDK_99.99_) of bacterial cells in the population for BAC (benzalkonium chloride)- and DADMAC (dimethylammonium chloride)-exposed isolates compared to the control.

The tolerance of the biocide-exposed isolates was measured as the minimum duration for killing 99.99% (MDK_99.99_) of the BAC and the DADMAC-exposed isolates when treated with a very high ampicillin concentration. There was a decrease in the initial bacterial concentration of 2.5 × 10^8^ CFU/mL to 2.5 × 10^4^ CFU/mL on the second day, giving an MDK_99.99_ of 30 h for BAC 12 (Figure 1). For DADMAC-exposed isolates, the bacterial count dropped from 1.8 × 10^8^ to 1.5 × 10^4^ CFU/mL, giving an MDK_99.99_ value of 28 h (Figure 1). There was no reduction in the bacterial count of the controls.

#### 3.1.2. Tolerance among Antibiotics Residue Exposed Isolates

**Figure 2:**
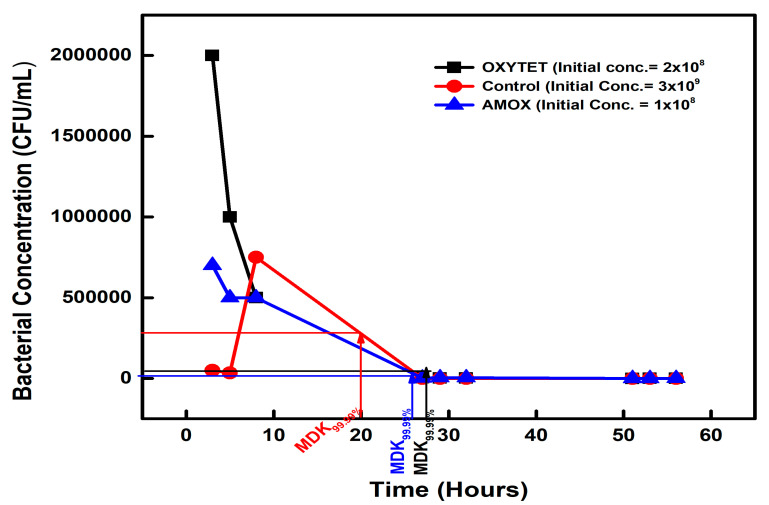
The minimum time required to kill 99.99% (MDK_99.99_) of the AMX (amoxycillin)- and OXYTET (oxytetracycline)-exposed isolates compared to the control.

The microbial count of the amoxicillin-exposed isolates decreased from 1 × 10^8^ CFU/mL to 1 × 10^4^ CFU/mL on the second day, when exposed to a very high ampicillin concentration, giving an MDK_99.99_ of 26 h (Figure 2). The OXYTET-exposed isolates had an MDK_99.99_ value of 28 h, with the initial concentration of 2 × 10^8^ CFU/mL decreasing to 2 × 10^4^ CFU/mL on the second day (Figure 2).

#### 3.1.3. Tolerance among Metal-Exposed Isolates

**Figure 3:**
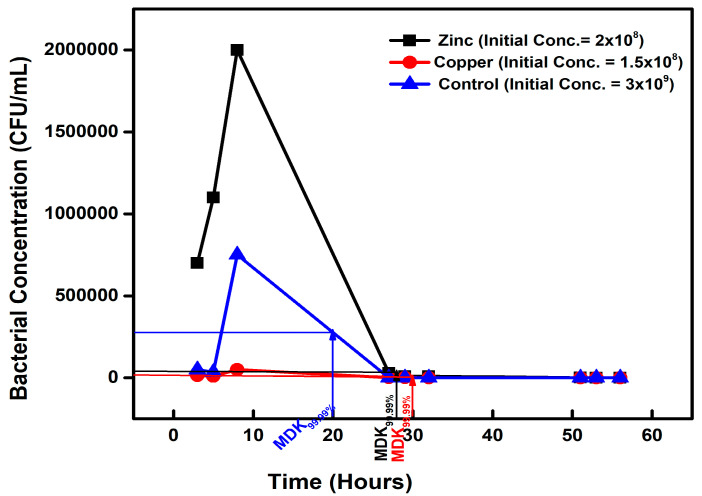
The minimum time required to kill 99.99% (MDK_99.99_) of the zinc- and copper-exposed isolates compared to the control.

When subjected to a high ampicillin concentration, the Zn- and Cu-exposed isolates had an MDK_99.99_ of 28 h and 30 h, with bacterial counts reducing from 2 × 10^8^ CFU/mL to 2 × 10^4^ CFU/mL and 1.5 × 10^8^ CFU/mL to 1.5 × 10^4^ CFU/mL on the second day, respectively (Figure 3).

#### 3.1.4. Tolerance for the Combined Chemical-Exposed Isolates

**Figure 4:**
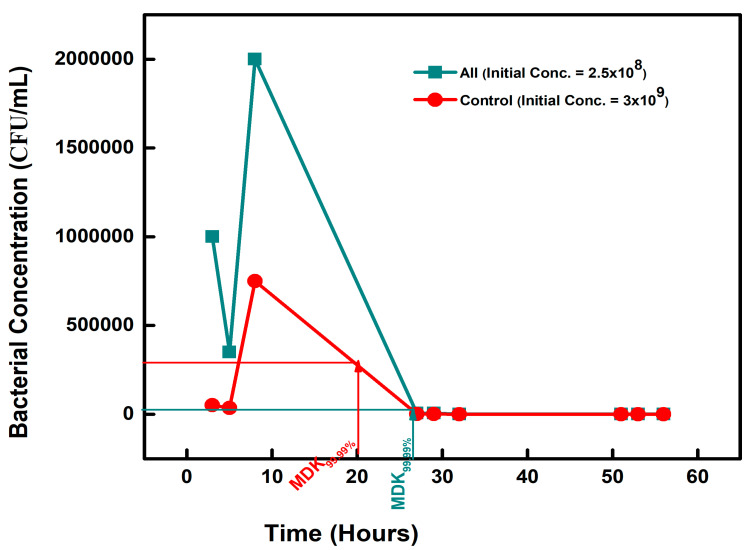
The minimum time required to kill 99.99% (MDK_99.99_) of the ALL (combined chemicals)-exposed isolates compared to the control.

The combined chemical (ALL)-exposed isolates subjected to a high ampicillin concentration recorded an MDK_99.99_ value of 26 h with a concentration of 2.5 × 10^4^ CFU/mL on the second day, from an initial 2.5 × 10^8^ CFU/mL (Figure 4).

#### 3.1.5. Comparison of Pollutant-Treated Isolates to Control in the Determination of the MDK_99.99_

The MDK_99.99_ of all the pollutant-exposed isolates, compared to that of the control, are summarised in Table 1. Compared with the control with an initial bacterial count of 3.5 × 10^9^ and an MDK_99.99_ of 20 h, the BAC-exposed isolates showed a 50% increase in the duration of killing, while the DADMAC-exposed isolates recorded a 40% increase following treatment with a very high ampicillin concentration. Also, for the AMX and OXYTET-exposed isolates, the MDK_99.99_ increased by 30% and 40%, respectively, after high ampicillin exposure. Furthermore, there was a 40% and 30% increase in the MDK_99.99_ for Zn and Cu-exposed isolates, respectively, compared to the control. Finally, ALL-treated isolates displayed a 30% increase in the MDK_99.99_ value compared to the control upon treatment with high antibiotic concentrations.

### 3.2. Determination of the Mutant Selection Window

The MSW of the isolates exposed for 30 days to the different environmental stressors was obtained by determining the MIC and MPC of these isolates. The average MSWs for the various isolates, compared to the control, are presented in Table 2.

No significant increase in the MSW was observed for the OXYTET treatment compared to the control. For the AMX treatment, no significant differences were observed in the MSW of the AMX, OXYTET, and Cu-exposed isolates compared with the control. However, there was a 41.67% increase in the MSW of Zn-exposed isolates and a 100% increase in the MSW for the DADMAC, BAC, and ALL-exposed isolates. Finally, ampicillin treatment revealed a 100% (Zn-exposed isolates) and 300% (ALL-exposed isolates) increase in the MSW of the test isolates compared to the control (Table 3).

## 4. Discussion

The present study investigated the impact of exposure to different environmental pollutants on the development of resistance in bacteria, using *E. coli* as a model organism. Previously exposed isolates were treated with extremely high ampicillin concentrations, and the time to reduce their population by 99.99% was determined. It was observed that exposure to environmental concentrations of biocides, antibiotics, and heavy metals induced tolerance in the test organism. This was demonstrated by an increase in the time required to kill 99.99% of the initial count of the exposed bacterial population and a broadening of the mutant selection window.

### 4.1. Tolerance in Exposed Isolates

Tolerance develops when the number of persister cells in a bacterial population increases depending on prevailing conditions [2,4,5,9]. This means that the increase in the number of persister cells should translate to an increase in tolerance, indicated by an increase in the MDK_99.99_ of the isolates. This is triggered by the expression of genes, as seen in the 30-day-exposed fully susceptible *E. coli* in the current study. Such gene expression is a first step towards resistance, as further exposure may lead to the development of resistance genes. Furthermore, this shows that tolerance enables the bacteria to survive stress, which if not eliminated, lowers the bacterial fitness cost for selecting and expressing resistance genes [24,29,30].

The current experiment reveals that exposing *E. coli* to sub-inhibitory concentrations of different chemicals can increase the time required to kill the exposed isolates (MDK_99.99_). This observation indicates that the exposed isolates may survive longer in the environment compared to unexposed cells (in this case, the control). From the results, BAC 12 had the highest MDK_99.99_, 50% higher than the control, while amoxicillin and copper had the lowest MDK_99.99_ (30% higher than the control). In an earlier experiment on these isolates, whole genome sequencing showed mutations in the isolates after 30 days of exposure, with no phenotypic resistance [20]. However, this study identified more survivors in the exposed isolates compared to the control isolates, despite the exposure to a very high ampicillin concentration (MIC × 25). This indicates a probable adaptation to antimicrobials through tolerance, as there was an increase in MDK instead of the MIC [24].

Most of the genes detected through WGS/SNP (*acnB, cusA, degQ, epmA, queG, hsmp, mlc, murP, nudK, ptsG, purH, queG, robA, srlE, tsaB, yddG* and *yqhH*) [20] are involved in the repression of oxidative stress, SOS-dependent gene repairs, toxin/antitoxin efflux actions, skin permeability, biofilm formation, or cellular physiological processes. These are factors mostly employed by bacteria for tolerance and persister cell production. [31,32].

Isolates exposed to BAC 12 had the highest MDK_99.99_. In addition to the genes above, these isolates also harboured the *fliL* *gene*. The *fliL* gene was only detected in BAC 12 and oxytetracycline isolates, which may have contributed to the high MDK_99.99_ observed in these two isolates. *fliL* is one of the seven genes within the flagellar-associated *flaA* locus that works with specific proteins to increase bacteria motility [33,34]. Cell motility contributes to bacteria survival and virulence, and survival due to motility does not increase the MIC of the survivors [35]. This result agrees with previous studies indicating that the exposure of *E. coli* to sub-MIC concentrations of BAC resulted in the expression of genes associated with efflux, outer membrane porins and motility, increasing tolerance to BAC [36,37,38,39]. Therefore, the detected *filL* gene likely contributed to increased tolerance to stressors in the BAC 12 and OXYTET-exposed isolates, demonstrated by the increased MDK when compared to control.

Oxidative stress, which results from over-accumulating reactive oxygen species (ROS) (produced by normal metabolism and essential for cell signalling and homeostasis), leads to DNA damage and cell death. For example, the *mut*M and *Fpg* (formamidopyrimidine glycolase) genes were only detected in zinc-exposed isolates (with a 40% increase in tolerance compared to the control). *Fpg* is a bifunctional DNA glycosylase that cleaves the N-glycoside bond of redox-damaged purines and incises the phosphodiester backbone to yield single-strand breaks with 3′ and 5′phosphoryl ends [40]. In repairing oxidative-damaged DNA, *mut*M is the primary DNA glycosylase that removes the oxidised purines and some pyrimidines [40]. As such, it is actively involved in the repair of lesions in the transcription of intermediates [41,42,43]. The repair of genetic materials is part of the SOS response, which in *E. coli*, contributes to the transcription of genes involved in DNA repair, the production of persister cells, biofilm formation, and tolerance mechanisms [31,44,45].

Another gene only detected in DADMAC isolates was *omp*D, a major porin protein in the outer membrane of cells, involved in the efflux of toxins/antitoxins through the cell membrane, which is very important in tolerance [46,47,48]. Furthermore, the *nud*K gene, also known as GDP-mannose hydrolase (which was expressed by other isolates except for DADMAC-exposed isolates), is a member of the ADP-ribose pyrophosphate sub-family of the *Nudix* hydrolases, and promotes biofilm formation, contributing to persister production and tolerance [31,49]. In addition, the *hsmP* gene, also detected in all the isolates, encodes for biofilm formation [50]. Biofilm formation is very important for tolerance as it encourages the production of persister cells within the population.

Another gene detected in all the isolates was the *mur*P gene. This gene contributes to tolerance by encoding the permease component of the N-acetylmuramic acid PTS transport system, facilitating the uptake and transportation of anhydrous acetylmuramic (*anyMurNAc*) acid. In addition, it encodes *anm*K (anhydro-N-acetyl muramic acid kinase), which is needed to convert imported *anhMurNA*c to *MurNAc-P*, a carbon and energy source for *E. coli*. The cAMP and catabolic response genes in *E. coli* negatively relay *rpoS*, so the over-expression of *rpoS* induces stationary phase cells and persister production and increases tolerance to antimicrobials [31,51,52,53,54]. *acnB* is also similar [55,56,57].

During exposure to antimicrobials, tolerant bacteria can withstand antimicrobial exposure and resume growth and virulence once the stressor is removed; hence their ability to stay alive in fluctuating exposure to antimicrobials, especially above the MIC [19]. This is facilitated by *degQ,* a serine endoprotease and a homologous member of the *HtrA* (high-temperature requirement A) protein family with *degP* and *degS*, which is involved in the degradation of transient proteins, stress sensing, regulation, and protection during unfolded protein responses, especially in isolates exposed to high temperature and nutrient deprivation [31,54,58].

Mutations that lead to tolerance are essential for the continued survival of the bacterial population, as the increased number of persisters creates a reservoir of non-susceptible bacteria, enabling these bacteria to survive antimicrobials at bactericidal concentrations [24]. Therefore, tolerance allows bacteria to adapt to adverse conditions for extended periods; if conditions continue, they select resistance genes with a decreased fitness cost. This can be attributed to the fact that persister cells naturally develop resistance genes, although these genes are usually lost due to high fitness cost. However, tolerance could lead to an increase in persister cells that can easily spread these genes within the population, leading to pseudo resistance. Therefore, tolerance has been observed as the first step towards resistance in most bacteria, especially in environments polluted with very high antimicrobial concentrations [29,59,60].

### 4.2. Effect of Exposure on the Mutant Selection Window

The MIC and MPC results showed that of the 30-day Cu and Zn-exposed isolates, only Zn-exposed isolates had an increase in their MSW to the tested antibiotics, compared to the unexposed control. On the other hand, there was a significant increase in the MSW of amoxicillin and ampicillin for the Zn-exposed isolates, while the MSW of OXYTET remained the same. This signifies that the exposure of susceptible bacteria to sub-MIC concentrations of heavy metals like Zn in the environment may contribute to the development of more persister cells within the population. These non-susceptible cells could outgrow susceptible ones in the population, increasing the chances of antimicrobial resistance development and spread in bacterial population [28,61,62]. This also agrees with Fridman et al. [24] and Levin-Reisman et al. [60], who stated that tolerant strains enhance the population’s survival and extend their survival window beyond the MPC, thereby increasing the MSW.

For the biocides, BAC-exposed isolates exhibited the most significant increase in the MSW compared to the control, especially when tested against amoxicillin and ampicillin but not with oxytetracycline. This shows that biocides in the environment, like heavy metals, can also trigger selection pressures for tolerant strains, contributing to the further resistance of the isolates against known antimicrobials and facilitating mutation [28,61,62].

Unlike the heavy metals and biocides tested in the current study, the antibiotic-exposed isolates all had similar results, indicating only a slight increase in the MSW following ampicillin treatment. This suggests that antibiotics in the environment may not be major contributory factors for increased antimicrobial resistance [12]. This agrees with a previous study which stated that antibiotics in the environment exert less selection pressure for antimicrobial resistance than other stressors, such as heavy metals [62].

## 5. Conclusions

Although several studies have reported that the exposure of environmental bacteria to different antimicrobials would lead to the development of resistance, this has mostly been undertaken using unrealistically high concentrations of the stressors. The present study revealed that exposing *E. coli* to different chemicals at environmental concentrations for 30 days triggered increased tolerance in the bacterial population when exposed to a high ampicillin concentration. This affected the MSW of the isolates against amoxicillin and ampicillin treatment. Survival in the presence of antimicrobials, even above the MIC value, can be attributed to the emergence of more persister cells, hence increased tolerance, and this contributed to the observed differences in the MSW of the exposed isolates compared to controls that were not exposed to any chemical. Given the complexity of the environmental dimension of AMR, these results call for a deeper analysis of the mechanisms influencing antimicrobial resistance in the environment. It should, however, be noted that the current study was conducted under static physicochemical conditions and that other factors like temperature, pH, and organic substances present in the natural environment were not investigated. Nevertheless, the results call for greater attention to the release of antimicrobials in the environment; these could have severe negative ecological and public health consequences, especially in resource limited areas without access to basic sanitation facilities and potable water supplies.

## Figures and Tables

**Table 1:** Summary of the tolerance experiment results showing the initial concentrations, final concentrations, MDK_99.99_, and percentage difference compared to the control.

Chemical-Exposed Isolate	Initial Concentration (CFU/mL)	Concentration (CFU/mL) on Day 2	MDK_99.99_	Percentage Difference
BAC 12	2.5 × 10^8^	2.5 × 10^4^	30	+50%
DADMAC 10	1.5 × 10^8^	1.5 × 10^4^	28	+40%
AMOXICILLIN	1 × 10^8^	1 × 10^4^	26	+30%
OXYTETRACYCLINE	2 × 10^8^	2 × 10^4^	28	+40%
ZINC	2 × 10^8^	2 × 10^4^	28	+40%
COPPER	1.5 × 10^8^	1.5 × 10^4^	26	+30%
CONTROL	3 × 10^9^	3 × 10^5^	20	0

BAC = benzalkonium chloride; DADMAC = dimethylammonium chloride; MDK = minimum duration of killing; CFU = colony forming units.

**Table 2:** Mean mutant selection window values for the ampicillin-, oxytetracycline-, and amoxicillin-treated isolates.

MSW Treatment	Mean MSW	Standard Deviation
Ampicillin	266.86	27.67
Ampicillin control	144.766	8.76
Oxytetracycline	254.76	0.12
Oxytetracycline control	255.45	0.17
Amoxycillin	452.16	42.84
Amoxycillin control	348.57	59.02

**Table 3:** Mutant selection window of the 30-day-exposed isolates following oxytetracycline, amoxicillin and ampicillin treatment.

MSW Treatment	Exposure Pollutant	Test Isolates			Controls		
		MIC	MPC	MSW	MIC	MPC	MSW
OXYTETRACYCLINE	AMX	2	256	0.5–256	2	256	2–256
	OXYTET	2	256	2–256	2	256	2–256
	COPPER	2	256	2–256	2	256	2–256
	ZINC	2	256	2–256	2	256	2–256
	BAC	2	256	2–256	2	256	2–256
	DADMAC	0.5	256	0.5–256	0.5	256	0.5–256
	ALL	2	256	2–256	2	256	2–256
AMOXICILLIN	AMX	8	512	8–512	8	512	8–512
	OXYTET	8	512	8–512	8	512	8–512
	COPPER	8	512	8–512	8	512	8–512
	ZINC	8	512	8–512	8	256	8–256
	BAC	8	512	8–512	8	256	8–256
	DADMAC	8	256	8–256	8	128	8–128
	ALL	8	512	8–512	8	256	8–256
AMPICILLIN	AMX	8	256	8–256	8	256	8–256
	OXYTET	8	256	8–256	8	256	8–256
	COPPER	8	256	8–256	8	256	8–256
	ZINC	8	256	8–256	8	128	8–128
	BAC	8	512	8–512	8	128	8–128
	DADMAC	8	256	8–256	8	256	8–256
	ALL	8	258	8–258	8	128	8–128

MSW = mutant selection window; MPC = mutant prevention concentration; MIC = minimum inhibitory concentration; BAC = benzalkonium chloride; DADMAC = dimethylammonium chloride; OXYTET = oxytetracycline; AMX = amoxycillin.

## Data Availability

All data have been added to the manuscript. Any further data would be provided by the authors upon responsible request.
